# Development and Validation of a Nomogram for Predicting Survival in Gallbladder Cancer Patients With Recurrence After Surgery

**DOI:** 10.3389/fonc.2020.537789

**Published:** 2021-01-11

**Authors:** Mingyu Chen, Shijie Li, Win Topatana, Xiaozhong Lv, Jiasheng Cao, Jiahao Hu, Jian Lin, Sarun Juengpanich, Jiliang Shen, Xiujun Cai

**Affiliations:** ^1^ Department of General Surgery, Sir Run-Run Shaw Hospital, Zhejiang University School of Medicine, Hangzhou, China; ^2^ Engineering Research Center of Cognitive Healthcare of Zhejiang Province, Hangzhou, China; ^3^ Zhejiang University School of Medicine, Hangzhou, China; ^4^ Department of General Surgery, First People’s Hospital, Mudanjiang, China; ^5^ Department of General Surgery, Longyou People’s Hospital, Quzhou, China

**Keywords:** gallbladder cancer, recurrence, survival, nomogram, prognostic model

## Abstract

**Background:**

The management of gallbladder cancer (GBC) patients with recurrence who need additional therapy or intensive follow-up remains controversial. Therefore, we aim to develop a nomogram to predict survival in GBC patients with recurrence after surgery.

**Methods:**

A total of 313 GBC patients with recurrence from our center was identified as a primary cohort, which were randomly divided into a training cohort (N = 209) and an internal validation cohort (N = 104). In addition, 105 patients from other centers were selected as an external validation cohort. Independent prognostic factors, identified by univariate and multivariable analysis, were used to construct a nomogram. The performance of this nomogram was measured using Harrell’s concordance index (C-index) and calibration curves.

**Results:**

Our nomogram was established by four factors, including time-to-recurrence, site of recurrence, CA19-9 at recurrence, and treatment of recurrence. The C-index of this nomogram in the training, internal and external validation cohort was 0.871, 0.812, and 0.754, respectively. The calibration curves showed an optimal agreement between nomogram prediction and actual observation. Notably, this nomogram could accurately stratify patients into different risk subgroups, which allowed more significant distinction of Kaplan-Meier curves than that of using T category. The 3-year post-recurrence survival (PRS) rates in the low-, medium-, and high-risk subgroups from the external validation cohort were 53.3, 26.2, and 4.1%, respectively.

**Conclusion:**

This nomogram provides a tool to predict 1- and 3-year PRS rates in GBC patients with recurrence after surgery.

## Introduction

Gallbladder cancer (GBC) is a rare tumor with an estimated annual incidence of 3 individuals every 100,000 people, but it remains the most common and aggressive malignancy of the biliary tract associated with poor prognosis (1–4). Surgical resection is regarded as one of the most effective treatments for GBC (5). Unfortunately, even with radical resection, more than 60% of patients will suffer from postoperative recurrence within five years (6). The high rates of recurrence and metastasis cause high mortality and the five-year survival rate is under 5% (2, 6).

Several individual predictive models were performed to evaluate survival for GBC patients after surgery in previous studies (7–9). Those nomograms were constructed based on variables limited to primary tumor characteristics and preoperative clinical factors. However, for GBC patients with recurrence, postoperative deaths are mainly caused by intrahepatic recurrence following by liver failure or distant metastasis resulting in cancer-associated cachexia (10). Moreover, post-recurrence survival (PRS) is greatly influenced by the management strategies for recurrence and clinical factors at recurrence rather than those features of the primary tumor. Therefore, previous nomograms may not achieve accurate expectations regarding the evaluation of survival in GBC patients with recurrence. Consequently, there is a reasonable and strong need for a post-recurrence model to assist clinicians in patient management.

In this study, we aim to develop a nomogram for the prediction of PRS in GBC patients with recurrence after curative resection, whereby an independent cohort from other medical centers was selected for external validation.

## Material and Methods

### Study Population and Design

**Figure 1:**
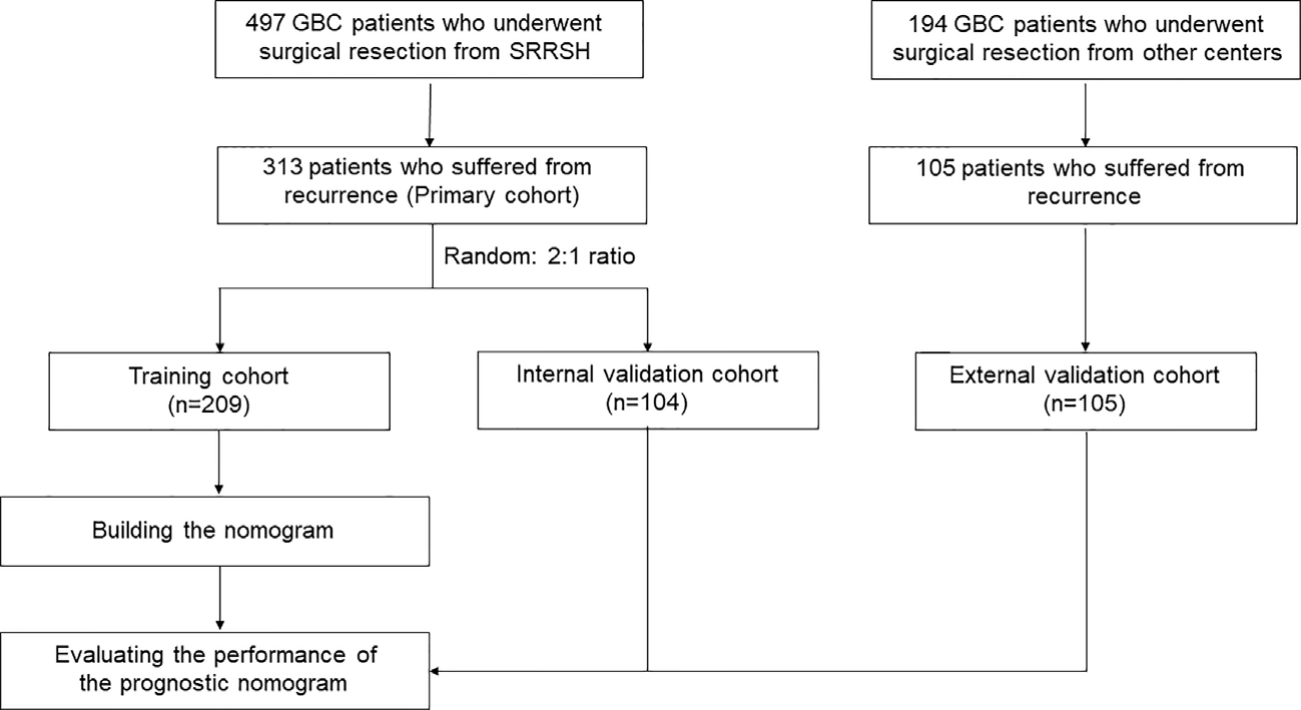
Flowchart of population selection and study design.

A total of 497 GBC patients who received treatment between January 2005 and December 2014 from Sir Run-Run Shaw Hospital were selected according to the following inclusion criteria: (1) patients undergo surgical resection with negative margins; (2) no history of other malignancies; and (3) follow-up was available and more than 3 months. A total of 313 GBC patients with recurrence after surgery were identified as a primary cohort. The primary cohort was randomly divided into a training cohort (N = 209) and an internal validation cohort (N = 104) in a 2:1 ratio. Finally, an independent cohort consisting of 105 patients from other medical centers were used for external validation. The flowchart was summarized in **Figure 1**. Ethical approval was obtained from each medicine institutional review board; Each patient’s informed consent was available.

A standardized data form was created to collect relevant information as follows: (1) demographic data (age, gender, body mass index, smoking, diabetes, jaundice, family history of GBC, stone); (2) tumor markers [carbohydrate antigen 19-9 (CA19-9), carcinoembryonic antigen (CEA)]; (3) pathologic features (tumor size, tumor differentiation; T-stage, lymph nodes status); (4) treatment-related details [liver resection (yes/no), post-operative complications; (5) recurrent data (time-to-recurrence, site of recurrence, CA19-9 and CEA at recurrence, jaundice at recurrence, treatment of recurrent tumors]. Follow-up data were obtained from the most recent medical review or regular follow-up that was performed by our team every 3–6 months. Time-to-recurrence was defined from the surgery date to the date when the patient was confirmed as recurrence. Post-operative complications included bleeding, infection, liver failure, and so on.

### Statistical Analysis

Survival curves for each variable were estimated and compared *via* the Kaplan-Meier method and Cox regression, respectively. The identification of predictors was performed using a two-stage process (univariate analysis followed by multivariable analysis) because of the small sample size and the number of factors of interest. If the variable achieved significance at P less than 0.05, it would be taken into the multivariable analysis using the Cox regression model in SPSS 19.0 (SPSS, Chicago, IL). Only significant variables (P < 0.05) in multivariable analysis with the back-ward step-down process were identified as independent factors, which were selected to build our prognostic nomogram using R 3.6.0 (https://www.r-project.org) with the survival and rms package. The performance of this nomogram was evaluated by the concordance index (C-index) and calibration curves in the internal and external validation cohort, respectively.

The discrimination ability of the nomogram was analyzed by grouping the patients into different risk groups that were then used to plot the Kaplan-Meier curves. The cutoff values were determined to identify the risk subgroups according to the total risk scores related with the predicted survival from high to low in the training cohort. The cutoff values were then used in internal and external validation cohorts, and each Kaplan-Meier curve was performed. Moreover, the independent discrimination ability of the nomogram beyond TNM staging was illustrated by comparing Kaplan-Meier curves.

## Results

### Clinicopathologic Characteristics

The related clinicopathologic characteristics of GBC patients in the training (N = 209), internal validation (N = 104) and external validation (N = 105) cohort were listed in **Table 1**. In the training cohort, lymph nodes status details were unavailable for 13 patients (6.22%). Almost half of the patients with GBC recurrence underwent supportive care and died one year after recurrence. The 1- and 3-year PRS rate was 40.0 and 10.3%, respectively. Similar results were observed in the internal validation cohort. In the external validation cohort from other medical centers, the 1- and 3-year post-recurrence survival PRS rate was 46.7, and 13.5%, respectively.

**Table 1:** Clinical and pathologic characteristics of GBC patients.

Variables	Training cohort (n = 209)	Internal validation cohort (n = 104)	External validation cohort (n = 105)
Age			
≤60	84 (40.19%)	52 (50.00%)	39 (37.14%)
>60	125 (59.81%)	52 (50.00%)	66 (62.86%)
Gender			
Female	133 (63.64%)	74 (71.15%)	68 (64.76%)
Male	76 (36.36%)	30 (28.85%)	37 (35.24%)
BMI			
<23.9	62 (29.67%)	43 (41.35%)	28 (26.67%)
24–26.9	132 (63.16%)	51 (49.04%)	69 (65.71%)
> 27	15 (7.18%)	10 (9.62%)	8 (7.62%)
Smoking			
Yes	57 (27.27%)	23 (22.12%)	18 (17.14%)
No	152 (72.73%)	81 (77.88%)	87 (82.86%)
Diabetes			
Yes	39 (18.66%)	12 (11.54%)	17 (16.19%)
No	170 (81.34%)	92 (88.46%)	88 (83.81%)
Jaundice			
Yes	9 (4.31%)	7 (6.73%)	4 (3.81%)
No	200 (95.69%)	97 (93.27%)	101 (96.19%)
Family History of GBC			
Yes	6 (2.87%)	5 (4.81%)	7 (6.67%)
No	203 (97.13%)	99 (95.19%)	98 (93.33%)
Stone			
Yes	113 (54.07%)	51 (49.04%)	58 (55.24%)
No	96 (45.93%)	53 (50.96%)	47 (44.76%)
CA19-9			
<37 kU/L	99 (47.37%)	55 (52.88%)	49 (46.67%)
≥37 kU/L	110 (52.63%)	49 (47.12%)	56 (53.33%)
CEA			
<5 ng/ml	151 (72.25%)	77 (74.04%)	72 (68.57%)
≥5 ng/ml	58 (27.75%)	27 (25.96%)	33 (31.43%)
Tumor size			
<1 cm	15 (7.18%)	18 (17.31%)	15 (14.29%)
1–3 cm	85 (40.67%)	44 (42.31%)	45 (42.86%)
3–5 cm	65 (31.10%)	29 (27.88%)	30 (28.57%)
> 5 cm	44 (21.05%)	13 (12.50%)	15 (14.29%)
T-stage			
T3	72 (34.45%)	27 (25.96%)	44 (41.90%)
T2b	78 (37.32%)	47 (45.19%)	36 (34.29%)
T2a	56 (26.79%)	28 (26.92%)	24 (22.86%)
T1	3 (1.44%)	2 (1.92%)	1 (0.95%)
Tumor differentiation			
Low	114 (54.55%)	45 (43.27%)	59 (56.19%)
moderate	53 (25.36%)	32 (30.77%)	25 (23.81%)
High	42 (20.10%)	27 (25.96%)	21 (20.00%)
Lymph nodes statue			
Negative	75 (35.89%)	43 (41.35%)	39 (37.14%)
Positive	121 (57.89%)	51 (49.04%)	58 (55.24%)
Unknown	13 (6.22%)	10 (9.62%)	8 (7.62%)
Liver resection			
Yes	193 (92.34%)	93 (89.42%)	98 (93.33%)
No	16 (7.66%)	11 (10.58%)	7 (6.67%)
Post-operative complications			
Yes	18 (8.61%)	10 (9.62%)	11 (10.48%)
No	191 (91.39%)	94 (90.38%)	94 (89.52%)
Time to recurrence			
<1 year	138 (66.03%)	59 (56.73%)	71 (67.62%)
1–3 year	45 (21.53%)	28 (26.92%)	22 (20.95%)
>3 year	26 (12.44%)	17 (16.35%)	12 (11.43%)
Site of recurrence			
Intrahepatic	120 (57.42%)	54 (51.92%)	55 (52.38%)
Extrahepatic	28 (13.40%)	24 (23.08%)	16 (15.24%)
Both	61 (29.19%)	26 (25.00%)	34 (32.38%)
CA19-9 at recurrence			
<37 kU/L	45 (21.53%)	27 (25.96%)	27 (25.71%)
≥37 kU/L	164 (78.47%)	77 (74.04%)	78 (74.29%)
CEA at recurrence			
<5 ng/ml	110 (52.63%)	56 (53.85%)	53 (50.48%)
≥5 ng/ml	99 (47.37%)	48 (46.15%)	52 (49.52%)
Jaundice at recurrence			
Yes	56 (26.79%)	33 (31.73%)	31 (29.52%)
No	153 (73.21%)	71 (68.27%)	74 (70.48%)
Treatment of recurrence			
Supportive care	103 (49.28%)	48 (46.15%)	57 (54.29%)
Re-resection	12 (5.74%)	7 (6.73%)	1 (0.95%)
Chemotherapy	29 (13.88%)	15 (14.42%)	15 (14.29%)
Radiotherapy	33 (15.79%)	19 (18.27%)	20 (19.05%)
Chemoradiotherapy	26 (12.44%)	11 (10.58%)	10 (9.52%)
Other	6 (2.87%)	4 (3.85%)	2 (1.90)

BMI, body mass index; GBC, gallbladder cancer; CA19-9, carbohydrate antigen 19-9; CEA, carcinoembryonic antigen.

### Independent Prognostic Factors

**Figure 2:**
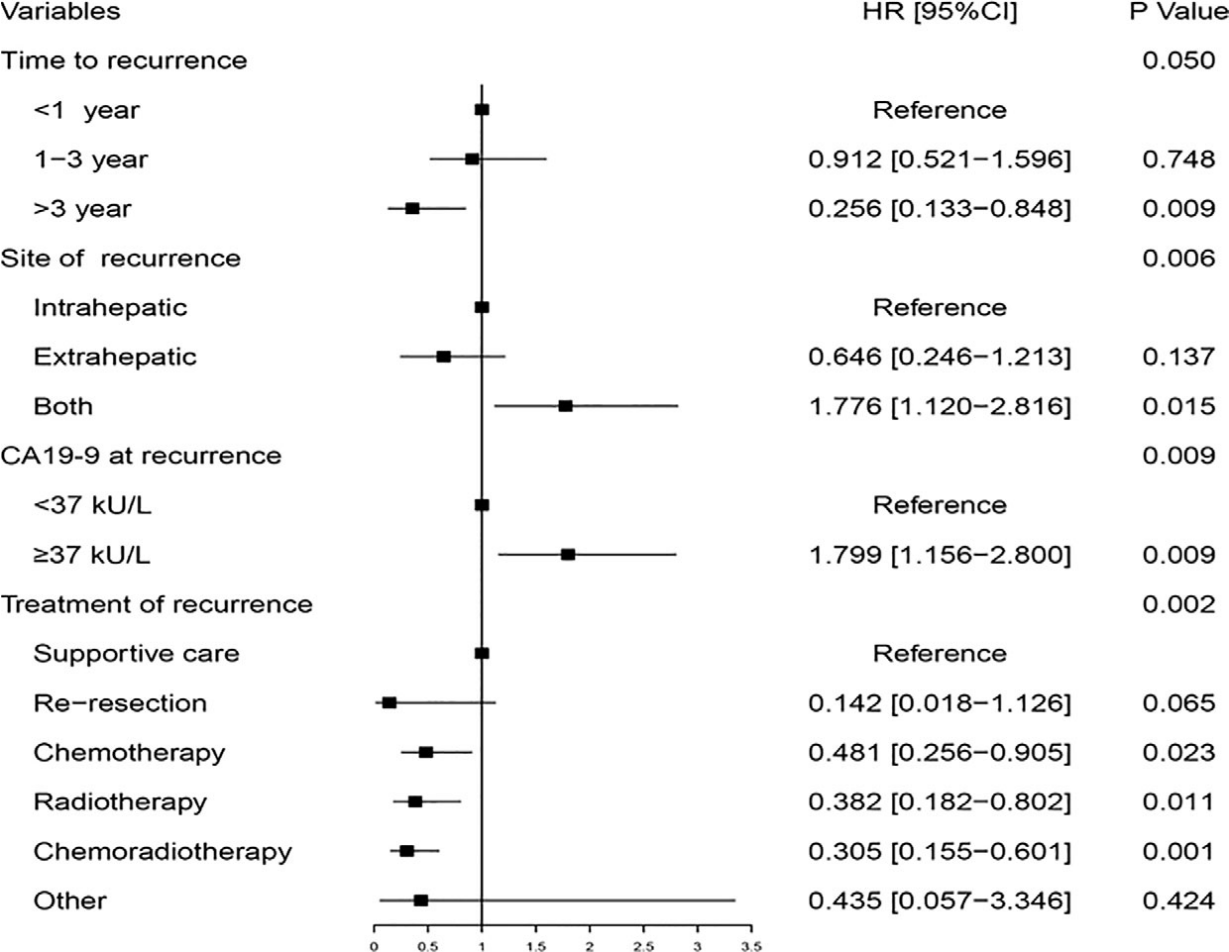
Multivariable analysis of the hazard ratio of post-recurrence survival in the training cohort.

Univariate analysis was performed to identify the significant factors related to PRS using the Kaplan-Meier method (**Supplemental Table S1**). Nine variables with P less than 0.05, including preoperative CA19-9, T-stage, positive lymph nodes, time-to-recurrence, site of recurrence, jaundice at recurrence, CA19-9 at recurrence, CEA at recurrence, and treatment of recurrence, were taken into multivariable analysis with the Cox regression model. The multivariable analysis determined that time-to-recurrence, site of recurrence, CA19-9 at recurrence, and treatment of recurrence were the four independent prognostic factors for PRS of GBC (**Figure 2**).

### Prognostic Nomogram

**Figure 3:**
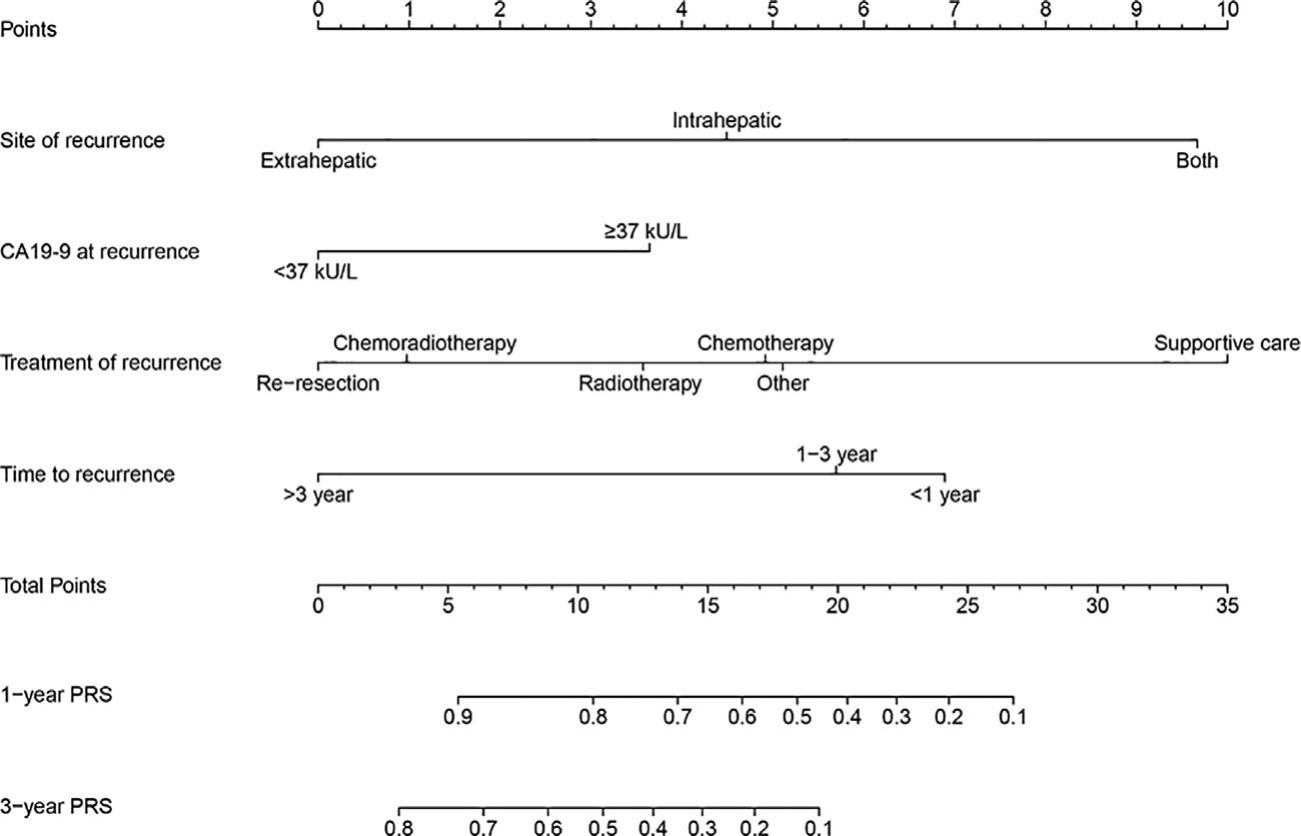
A prognostic nomogram for estimating the 1- and 3-year post-recurrence survival rates in the gallbladder cancer patients with recurrence after surgery.

Based on four independent prognostic factors, the nomogram was constructed to predict the 1- and 3-year PRS rates in GBC patients with recurrence after surgery (**Figure 3**). To use this nomogram, the total points from each point of the independent prognostic factor was added up to identify the predicted probability.

### Validation of the Nomogram

**Figure 4:**
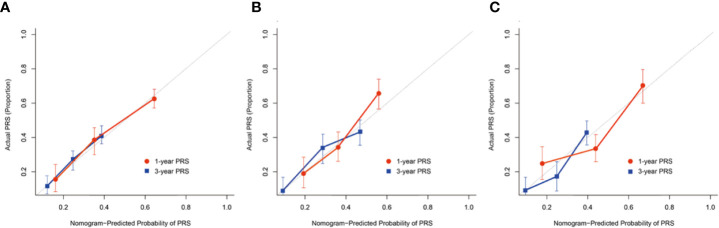
The calibration curves demonstrating how the 1- and 3-year post-recurrence survival predictions from the model compare to the actual observed survival in the **(A)** training, **(B)** internal, **(C)** external validation cohort.

The C-index and calibration curves were used to test and validate this prognostic nomogram. The C-index of the training, internal and external validation cohort was 0.871, 0.812, and 0.754, respectively. The calibration plot for the probability of 1- and 3- year PRS demonstrated optimal agreement between the prediction by nomogram and actual observation among cohorts (**Figure 4**).

### Discrimination Ability of the Nomogram

**Figure 5:**
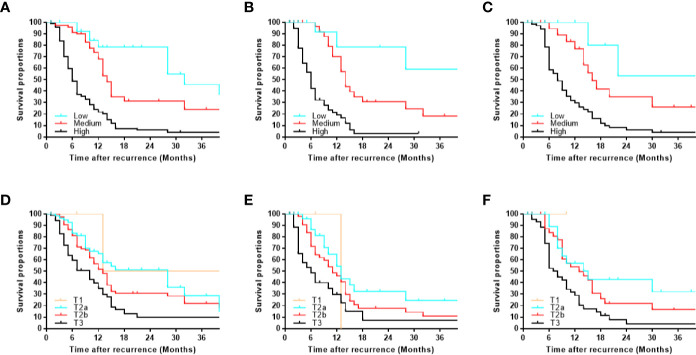
Kaplan-Meier post-recurrence survival curves for each risk subgroup of patients. Patients were stratified by our monogram in the **(A)** training, **(B)** internal, **(C)** external validation cohort. Patients were stratified by the T category in the **(D)** training, **(E)** internal, **(F)** external validation cohort.

According to sorting by the predicted PRS in the training cohort (**Supplemental Table S2**), the cutoff values (≤11, 11–17, >17) in the total risk scores were identified to divide the patients into three risk groups (low-, medium-, and high-risk subgroup), and each subgroup represented a distinct prognosis (**Figure 5A**). The median PRS in the low-, medium-, and high-risk subgroup was 32, 14, and 6 months, and the 3-year PRS rate was 45.7, 24.1, and 4.0%, respectively. Similar results were observed in the internal and external validation cohort (**Figures 5B, C**). Notably, after applying the cutoff values to group patients, stratification into different risk subgroups allowed more significant distinction between Kaplan-Meier curves for PRS than that of using T category (**Figures 5D–F**).

## Discussion

Gallbladder cancer is a rare malignancy that is usually a fatal disease. Even though surgical resection is regarded as the most effective treatment for GBC, most of the cases already became advanced or metastasized by the time it was diagnosed (11). Recurrence is fairly common among GBC after surgery, primarily resulting from the biological characteristics of high invasion and easily metastasis ability (12, 13). Compared to other solid cancers, the knowledge of biological characteristics of GBC is still limited. Available data on treatment of recurrence such as supportive care, re-resection, chemotherapy, radiotherapy, chemoradiotherapy, and target therapies would have the potential to improve patient prognosis after recurrence (14). However, there is little information available regarding survival evaluation in GBC patients with recurrence after surgery. To the best of our knowledge, our study is the first nomogram focusing on the post-recurrence survival of GBC. In this study, we have constructed and validated a prognostic nomogram for evaluating 1- and 3-year survival in GBC patients with recurrence after surgery based on four independent factors, including time-to-recurrence, site of recurrence, CA19-9 at recurrence, and treatment of recurrence.

The time-to-recurrence was regarded as an important and independent predictor of survival. The shorter time-to-recurrence in this study was associated with poorer prognosis. Margonis et al. (6) performed a postoperative GBC multi-center cohort and demonstrated that those patients with postoperative recurrence within 12 months or less had significantly worse survival rates. According to the results, around two-thirds of all recurrences occurred within the first 12 months following surgery. Similar rates (approximate 50–70%) of GBC recurrence at 1-year were reported in other medical centers (15, 16). Due to the short time-to recurrence or recurrence-free survival in the majority of GBC patients, therefore, evaluating postoperative recurrence survival is necessary and positive for GBC patient management.

In this study, the site of recurrence was divided into three types (intrahepatic, extrahepatic, and both). We found that those patients with both intrahepatic and extrahepatic recurrence commonly usually suffered from jaundice at recurrence and had a shorter survival time. Patients with jaundice were extremely complex, palliation is difficult to obtain because the place where infiltration occurs. On the other hand, jaundice must be solved before giving chemotherapy, which results in limited treatment strategies for them. Fortunately, intrahepatic recurrence is the most common recurrent site which occurred in more than half of patients. For patients with intrahepatic recurrence, more adjuvant therapies were available, including re-resection (17), chemotherapy (18), radiotherapy (19), chemoradiotherapy (20). For example, re-resection is not accessible to all populations, but to the patients with locoregional recurrence or a single tumor where surgery can be performed again (21). The importance of treatment of recurrence cannot be ignored. Our finding revealed that additional treatment for recurrence (e.g. re-resection, chemotherapy, radiotherapy, and chemoradiotherapy) could prolong the survival in GBC patients compared to patients who received supportive care. The similar results were reported that among GBC patients with recurrence could benefit from chemoradiotherapy (20, 22). Therefore, it could be speculated that the site of recurrence and treatment of recurrence were correlated with the survival in GBC patients with recurrence after surgery.

CA19-9 is one of the most common tumor markers used to evaluate the prognosis for patients with GBC (23, 24). Various studies have demonstrated that an elevated CA19-9 level is correlated with a poor prognosis in GBC patients (25–27). The study by Wen et al. (26) indicated that patients with CA19-9 level within the normal range had the best prognosis while patients with an elevated CA19-9 level had the poorest prognosis. Yamashita et al. (27) reported that non-normalization of CA19-9 level after resection of biliary tract cancer with curative intent was associated with worse overall survival, and postoperative CA19-9 level was superior to the preoperative CA19-9 level in the evaluation of survival in the GBC patients with recurrence after surgery. Our results also suggested that the patients with high CA19-9 at recurrence were associated with worse disease-specific and overall mortality.

This nomogram allowed us to divide GBC patients into the low-, medium- and high-risk subgroup with long-, medium-, and short-survival, respectively. In this study, we choose the cutoff value based on the corresponding quarter proportion of 3-year PRS (0–25, 25–50, 50–75, 75–100%) at first. Due to the patients in the subgroup of 3-year PRS >75% less than 10 patients (< 5%), we combined subgroup (50–75%) and subgroup (75–100%) as low-risk subgroup (3-year PRS ≥50%). Besides, the 3-year PRS = 25% corresponds to the total risk score is not an integer. In order to easily used in clinical practice, we choose 17 as one of the cutoff values in total risk score, which corresponding to the 3-year PRS = 20%. Therefore, the low-, medium- and high-risk subgroup is sorting by the predicted PRS according to 3-year PRS ≥50%, between 20–50%, and <20%, corresponding to the cutoff value (≤11, 11–17, >17) in the total risk scores. Most importantly, each final subgroup represented a distinct prognosis. To the best of our knowledge, there are no specialist scoring or grading for the management of GBC patients with recurrence except the TNM category (28, 29). Interestingly, we found that after applying the cutoff values to group patients, stratification into different risk subgroups allowed more significant distinction between Kaplan-Meier curves for survival than that of the T category. This demonstrated that the identification of different subgroups using our nomogram was superior to the conventual subgroups *via* the T category, which might have a positive impact on the treatment or care option. The selection of GBC patients with recurrence who need additional therapy or intensive follow-up remains controversial. Our nomogram could help clinicians to address such issues and provide individual decision-making. Additionally, our nomogram could provide more information on patient selection for designing clinical trials in the future.

Several limitations should be noted in this study. Most importantly, the major limitations were the small sample size, recall and treatment bias between the different medical centers. In the study, nearly half of the patients in all cohorts elected to have supportive care, one of the treatments of recurrence, at the time of recurrence, which likely affected the relative predictive value of our nomogram. The sample size was relatively small for the estimation of a predictive model. Thus, our findings can be used as starting point for further investigations. Further, the small sample size and the number of factors of interest led to the use of a sub-optimal process for the identification of predictors (univariate analysis followed by multivariable analysis), hence caution is suggested in the interpretation of our results. In addition, there was a large amount of fluctuation in the number of harvested lymph nodes (1 to 24) and the average number of harvested lymph nodes was 4.39, which were lower than the total number of harvested lymph nodes recommended by the AJCC 8^th^ edition guideline at least 6 (30, 31). For this reason, we applied the lymph node status (positive or negative) rather than the AJCC 8^th^ edition N category. Fortunately, our results were not affected by the usage of the possible classification based on the 8^th^ edition N category (**Supplemental Table S3**).

In conclusion, we have developed a prognostic nomogram for predicting the 1- and 3-year survival rate in the GBC patients with recurrence after surgery. The internal and external validation results revealed the high-performance level of this nomogram. However, considering the small sample size, the results of the nomogram is only a suggestion for future developments.

## Data Availability Statement

The datasets generated for this study are available on request to the corresponding authors.

## Ethics Statement

The studies involving human participants were reviewed and approved by the SRRSH of Medicine Institutional Review Board. The patients/participants provided their written informed consent to participate in this study.

## Author Contributions

MC and XC conceptualized and designed the study. XL, SL, JC, SJ, and JH collected and assembled the data. MC, WT, and XL analyzed and interpreted the data. All authors contributed to the article and approved the submitted version.

## Funding

This work was supported by the National Natural Science Foundation of China (Nos. 81827804, and 81800540), Zhejiang Provincial Natural Science Foundation of China (No. LQ18H160003), and Scientific Research Fund of Zhejiang Provincial Education Department (Y201941406).

## Conflict of Interest

The authors declare that the research was conducted in the absence of any commercial or financial relationships that could be construed as a potential conflict of interest.

## Supplementary Material

Supplementary Table 1 Univariable analysis of post-recurrence survival in the training cohort.

Supplementary Table 2 Points assignment and risk scores.

Supplementary Table 3 Univariable and multivariable analysis of post-recurrence survival using the possible N category in the training cohort.
