# Supplementary material for: Development and Validation of a Nomogram for Predicting Survival in Gallbladder Cancer Patients With Recurrence After Surgery
Source: Front Oncol. 2021 Jan 11;10:537789. doi: 10.3389/fonc.2020.537789 (PMC7829964; doi:10.3389/fonc.2020.537789)
Supplement: Supplementary Table 1 — Univariable analysis of post-recurrence survival in the training cohort. [file Table_1.docx]

Supplementary Material

| **Supplementary Table S1. Univariate analysis of post recurrence survival in the training cohort** | | |
| --- | --- | --- |
| Variables | HR (95%CI) | *P* |
| Age |  |  |
| ≤60 | Reference |  |
| >60 | 0.763 [0.507-1.148] | 0.194 |
| Gender |  |  |
| Female | Reference |  |
| Male | 1.065 [0.692-1.637] | 0.776 |
| BMI |  | 0.572 |
| <23.9 | Reference |  |
| 24-26.9 | 0.888 [0.570-1.385] | 0.601 |
| >27 | 0.687 [0.284-1.665] | 0.406 |
| Smoking |  |  |
| Yes | Reference |  |
| No | 0.582 [0.210-1.145] | 0.312 |
| Diabetes |  |  |
| Yes | Reference |  |
| No | 0.876 [0.320-2.394] | 0.796 |
| Jaundice |  |  |
| Yes | Reference |  |
| No | 0.938 [0.410-2.144] | 0.880 |
| Family History of GBC |  |  |
| Yes | Reference |  |
| No | 0.869 [0.310-2.412] | 0.789 |
| Stone |  |  |
| Yes | Reference |  |
| No | 0.987 [0.665-1.467] | 0.950 |
| CA19-9 |  |  |
| <37 kU/L | Reference |  |
| ≥37 kU/L | 1.554 [1.040-2.321] | 0.032 |
| CEA |  |  |
| <5 ng/mL | Reference |  |
| ≥5 ng/mL | 1.311 [0.854-2.012] | 0.216 |
| Tumor size |  | 0.534 |
| <1 cm | Reference |  |
| 1-3 cm | 0.716 [0.399-1.284] | 0.262 |
| 3-5 cm | 0.864 [0.472-1.582] | 0.636 |
| >5 cm | 1.066 [0.510-2.231] | 0.865 |
| T-stage |  | 0.007 |
| T3 | Reference |  |
| T2b | 0.612 [0.395-0.948] | 0.028 |
| T2a | 0.379 [0.208-0.690] | 0.001 |
| T1 | 0.410 [0.057-2.978] | 0.378 |
| Tumor differentiation |  | 0.126 |
| Low | Reference |  |
| moderate | 0.717 [0.437-1.178] | 0.189 |
| High | 0.593 [0.337-1.045] | 0.071 |
| Lymph nodes statue |  | 0.126 |
| Negative | Reference |  |
| Positive | 1.642 [1.051-2.565] | 0.029 |
| Unknown | 1.085 [0.473-2.491] | 0.847 |
| Liver resection |  |  |
| Yes | Reference |  |
| No | 0.821 [0.302-2.237] | 0.700 |
| Post-operative complications |  |  |
| Yes | Reference |  |
| No | 0.712 [0.339-1.496] | 0.370 |
| Time to recurrence |  | 0.005 |
| <1 year | Reference |  |
| 1-3 year | 0.662 [0.399-1.097] | 0.109 |
| >3 year | 0.250 [0.100-0.621] | 0.003 |
| Site of recurrence |  | 0.001 |
| Intrahepatic | Reference |  |
| Extrahepatic | 0.488 [0.321-0.742] | 0.001 |
| Both | 0.307 [0.137-0.687] | 0.001 |
| Jaundice at recurrence |  |  |
| Yes | Reference |  |
| NO | 0.462 [0.283-0.856] | 0.021 |
| CA19-9 at recurrence |  |  |
| <37 kU/L | Reference |  |
| ≥37 kU/L | 2.572 [1.540-4.296] | <0.001 |
| CEA at recurrence |  |  |
| <5 ng/mL | Reference |  |
| ≥5 ng/mL | 1.935 [1.294-2.895] | 0.001 |
| Treatment of recurrence |  | <0.001 |
| Supportive care | Reference |  |
| Re-resection | 0.095 [0.013-0.694] | 0.020 |
| Chemotherapy | 0.579 [0.321-1.045] | 0.070 |
| Radiotherapy | 0.306 [0.156-0.601] | 0.001 |
| Chemoradiotherapy | 0.340 [0.177-0.652] | 0.001 |
| Other | 0.279 [0.039-2.022] | 0.207 |
| BMI: body mass index; GBC: gallbladder cancer; CA19-9: carbohydrate antigen 19-9; CEA: carcinoembryonic antigen. | | |

| **Supplementary Table S2. Points assignment and risk scores.** | | |
| --- | --- | --- |
| Variables and total points | Scores | 3-year PRS |
| Time to recurrence |  |  |
| <1 year | 7 |  |
| 1-3 year | 5.7 |  |
| >3 year | 0 |  |
| Site of recurrence |  |  |
| Both | 9.7 |  |
| Intrahepatic | 4.5 |  |
| Extrahepatic | 0 |  |
| CA19-9 at recurrence |  |  |
| ≥37 kU/L | 3.7 |  |
| <37 kU/L | 0 |  |
| Treatment of recurrence |  |  |
| Supportive care | 10 |  |
| Other | 5.2 |  |
| Chemotherapy | 5 |  |
| Radiotherapy | 3.6 |  |
| Chemoradiotherapy | 1 |  |
| Re-resection | 0 |  |
| Total risk scores |  |  |
| 19 |  | 11 |
| 17 |  | 20 |
| 15 |  | 28 |
| 13 |  | 39 |
| 11 |  | 50 |
| 9 |  | 59 |
| 6 |  | 70 |
| 3 |  | 81 |
| PRS: postoperative recurrence survival; CA19-9: carbohydrate antigen 19-9. | | |

| **Supplementary Table S3.** Univariable and multivariable analysis of postoperative recurrence survival using the possible N category in the training cohort. | | | | | | |
| --- | --- | --- | --- | --- | --- | --- |
|  | Univariable analysis | |  |  | Multivariable analysis | |
| Variables | HR (95%IC) | P | | HR (95%IC) | | P |
| CA19-9 |  |  | |  | |  |
| <37 kU/L | Reference |  | |  | |  |
| ≥37 kU/L | 1.554 [1.040-2.321] | 0.032 | |  | |  |
| T-stage |  | 0.007 | |  | |  |
| T3 | Reference |  | |  | |  |
| T2b | 0.612 [0.395-0.948] | 0.028 | |  | |  |
| T2a | 0.379 [0.208-0.690] | 0.001 | |  | |  |
| T1 | 0.410 [0.057-2.978] | 0.378 | |  | |  |
| Lymph nodes statue |  | 0.034 | |  | |  |
| N0 | Reference |  | |  | |  |
| N1 | 1.376 [1.020-1.927] | 0.045 | |  | |  |
| N2 | 1.772 [1.151-2.865] | 0.005 | |  | |  |
| Unknown | 1.085 [0.473-2.491] | 0.847 | |  | |  |
| Time to recurrence |  | 0.005 | |  | | 0.048 |
| <1 year | Reference |  | | Reference | |  |
| 1-3 year | 0.662 [0.399-1.097] | 0.109 | | 0.881 [0.502-1.547] | |  |
| >3 year | 0.250 [0.100-0.621] | 0.003 | | 0.207 [0.080-0.537] | |  |
| Site of recurrence |  | 0.001 | |  | | 0.004 |
| Intrahepatic | Reference |  | | Reference | |  |
| Extrahepatic | 0.488 [0.321-0.742] | 0.001 | | 0.549 [0.247-1.220] | | 0.141 |
| Both | 0.307 [0.137-0.687] | 0.001 | | 1.823 [1.150-2.889] | | 0.011 |
| Jaundice at recurrence |  |  | |  | |  |
| Yes | Reference |  | |  | |  |
| NO | 0.462 [0.283-0.856] | 0.021 | |  | |  |
| CA19-9 at recurrence |  |  | |  | |  |
| <37 kU/L | Reference |  | |  | |  |
| ≥37 kU/L | 2.572 [1.540-4.296] | <0.001 | | 1.669 [1.077-2.586] | | 0.022 |
| CEA at recurrence |  |  | |  | |  |
| <5 ng/mL | Reference |  | |  | |  |
| ≥5 ng/mL | 1.935 [1.294-2.895] | 0.001 | |  | |  |
| Treatment of recurrence |  | <0.001 | |  | | 0.007 |
| Supportive care | Reference |  | | Reference | |  |
| Re-resection | 0.095 [0.013-0.694] | 0.020 | | 0.100 [0.012-0.720] | | 0.036 |
| Chemotherapy | 0.579 [0.321-1.045] | 0.070 | | 0.491 [0.261-0.924] | | 0.027 |
| Radiotherapy | 0.306 [0.156-0.601] | 0.001 | | 0.421 [0.202-0.879] | | 0.021 |
| Chemoradiotherapy | 0.340 [0.177-0.652] | 0.001 | | 0.309 [0.156-0.610] | | 0.001 |
| Other | 0.279 [0.039-2.022] | 0.207 | | 0.460 [0.060-3.552] | | 0.457 |
| BMI: body mass index; GBC: gallbladder cancer; CA19-9: carbohydrate antigen 19-9; CEA: carcinoembryonic antigen. | | | | | | |
